# Efficacy and safety of Yu-Ping-Feng powder for asthma in children: a protocol of systematic review and meta-analysis of randomized controlled trials

**DOI:** 10.1097/MD.0000000000018551

**Published:** 2020-01-03

**Authors:** Ruiyin Wang, Jianxin Wang, Jun Shu, Xianmin Gu, Hongwen Li, Yingxin Zi, Shufang Liu, Jiangtao Lin

**Affiliations:** a Beijing University of Chinese Medicine; b Respiratory and Critical Care Medicine, Center of Respiratory Medicine, China-Japan Friendship Hospital, National Clinical Research Center for Respiratory Diseases, Beijing, China.

**Keywords:** asthma, efficacy, protocol, systematic review, Yu-Ping-Feng

## Abstract

**Background:**

Asthma has become the most common chronic disease in children, which seriously affects children's health and growth. Yu-Ping-Feng powder (YPFP) is widely used for the treatment of asthma in children, but there are few meta-analyses to assess the add-on effects of YPFP in children with asthma. Therefore, it is necessary to conduct a systematic review to evaluate the efficacy and safety of YPFP in the management of asthma in children.

**Methods:**

PubMed, Cochrane Central Register of Controlled Trials (CENTRAL), EMBASE, Web of Science and the Chinese electronic databases including China Network Knowledge Infrastructure (CNKI), Chinese Biomedicine (CBM), Chinese Scientific Journals Database (VIP), and Wan Fang Database were searched for the randomized controlled trials (RCTs) of YPFP in children with asthma based on the eligibility criteria from the date of the database inception to 28 November 2018. Two reviewers assessed the articles and extracted data from the included RCTs independently. Data will be synthesized by either the fixed-effects or random-effects model according to a heterogeneity test. We will assess the risk of bias with the Cochrane Collaboration Tool and overall quality of the evidence using the Grading of Recommendations Assessment, Development and Evaluation system (GRADE). Primary outcomes include the improvement of symptoms including breathlessness, coughing, wheezing and the frequency of asthma exacerbations. Lung function, serum IgE level, blood eosinophil count, phlegm eosinophil count and adverse events will be assessed as the secondary outcomes. We will perform the data synthesis, sensitivity analyses, and subgroup analyses in the Rev-Man version 5.3 software. A funnel plot will be established to evaluate reporting bias.

**Results:**

This systematic review and meta-analysis will review and synthesis current clinical evidence of YPFP for the treatment of asthma in children.

**Conclusion:**

This analysis will provide high quality evidence of YPFP for the treatment of asthma in children.

**PROSPERO registration number:**

CRD42018111223.

## 1 Introduction

Bronchial asthma is one of the most common chronic airway inflammatory diseases, mainly involving a variety of inflammatory cytokines, such as mast cells and eosinophils, which is characterized by variable respiratory symptoms and variable airflow limitation.^[1]^ Asthma has become the most common chronic disease in children, which seriously affects children's health and growth.^[2]^ Recently, the prevalence of asthma in children in many countries and regions in the world is increasing and varies from country to country. For instance, the prevalence in childhood aged 6 to 7 years is from 6.7% in Indonesia to 37.6% in Costa Rica, while children aged 13 to 14 years from 3.4% in Albania to 31.2% in Isle of Man.^[3]^ In China, the prevalence has increased significantly over the past 20 years from 1.09% in 1990 to 3.02% in 2010.^[4]^ Invariably, costs for children with asthma were significantly greater than those for children without asthma. Total direct costs of pediatric asthma in the United States were US$5.92 billion in 2013 and average annual costs per child ranged from US$3076 to US$13612,^[5]^ accounting for 0.9% of Portugal's health care spending.^[6]^ In addition, the proportion of costs of children with acute asthma attacks and hospitalizations is still high in China, 77% and 47.3%, respectively.^[7]^

Clinicians advocate use of western medicine, symptomatic supportive treatment, to relieve symptoms, but the efficacy of this method seems not ideal. Asthma control is suboptimal in many children, especially in the Asia-Pacific region, the control rate of which is only 2.5% and uncontrolled up to 53.4%.^[8]^ Omalizumab as an addendum to standard therapies has been shown to be effective in children with severe allergic asthma, however, yielding incremental cost to increase the family burden.^[9–11]^

Therefore, it is imperative to develop a method that prevent the adverse effects of therapeutic agents, improve the efficacy of western medicine and reduce medical costs. The complex traditional Chinese prescription Yu-Ping-Feng powder (YPFP), consisting of parsnip, astragalus, sojutsu var, has widely been applied for the management of children's allergic asthma in China. Numerous clinical and experimental studies have shown that YPFP can improve asthma symptoms such as difficulty in breathing, tight airways, and coughing, significantly reduce the number of recurrent asthma attacks, and improve the quality of life of children with asthma. Besides, YPFP have shown to bidirectionally regulate the human immune system, inhibit the generation of IgE^[12–14]^ and suppress the expression levels of pro-inflammatory cytokines, TLR-4, NF-κB, and iNOS in the inflammatory models.^[15]^ Interestingly, harm on the normal immune system has not been found in the clinical studies when compared with western medicine.^[16,17]^

However, there are few meta-analyses to assess the add-on effects of YPFP in children with asthma. To evaluate the clinical efficacy and adverse effects of YPFP for the treatment of allergic asthma, we are writing the protocol to conduct a systematic review and meta-analysis using the available randomized controlled trials (RCTs).

## 2 Methods

### 2.1 Registration

The study protocol has already been registered on the international prospective register of the systematic review (PROSPERO) on 28 November 2018 (registration number: CRD42018111223). This systemic review and meta-analysis protocol followed the Preferred Reporting Items for Systematic Review and Meta-Analysis Protocols (PRISMA-P) guidelines.^[18]^

### 2.2 Eligibility criteria

#### 2.2.1 Types of studies

All randomized clinical trials (RCTs) that are assessed the efficacy and safety of YPFP for asthma in children were included in this review without language restrictions.

#### 2.2.2 Types of participants

Children (age < 18 years old) with asthma was eligible for inclusion regardless of the disease severity including mild, moderate, severe as well as difficult to treat asthma. There were no restrictions based on other conditions, such as gender, region, or Diagnostic criteria for asthma are based on the Chinese Childhood Asthma Prevention Guidelines^[4,19–23]^ or Global Strategy for Asthma Management and Prevention (GINA) Guidelines in different versions.^[24]^

#### 2.2.3 Types of interventions

Interventions included all types of YPPF alone and in combination with western medicine such as pills, capsules, Decoctions, and tablets, orally taken, for the treatment of allergic asthma in children. Both YPPF in combination with other Decoction and asthma with comorbidity were excluded. Other joint interventions such as acupuncture, cupping, moxibustion, massage, yoga, qigong, Tai Chi, and aromatherapy were also excluded.

#### 2.2.4 Types of outcomes

Primary outcomes were the improvement of symptoms including breathlessness, coughing, wheezing and the frequency of asthma exacerbations. The second outcomes included Lung function (forced expiratory volume in one second (FEV1) or predicted forced expiratory volume in one second (FEV 1%), FEV 1 /FVC (forced vital capacity)), serum IgE level, blood eosinophil count, phlegm eosinophil count. The safety outcomes were evaluated by adverse events.

### 2.3 Data sources and search strategy

The following electronic databases were searched from inception to 28 November 2018: PubMed, Cochrane Central Register of Controlled Trials (CENTRAL), EMBASE, Web of Science. We also searched for the Chinese electronic databases including China Network Knowledge Infrastructure (CNKI), Chinese Biomedicine (CBM), Chinese Scientific Journals Database (VIP), and Wan Fang Database. In addition, unpublished postgraduate papers in Chinese databases were searched. The search strategy for selecting the fields of title, abstract or keyword varied according to the characteristics of the databases. The search strategy for PubMed was listed as follows.

#1 Search (asthma [MeSH Terms] OR bronchial asthma [Title/Abstract] OR allergic asthma [Title/Abstract])
#2 Search (Yupingfeng [Title/Abstract] OR Yu Ping Feng San [Title/Abstract] OR Jade-Screen Powder [Title/Abstract])
#3 Search (children [Title/Abstract] OR child [Title/Abstract] OR kids [Title/Abstract] OR teenager [Title/Abstract])
#4 #1 and #2 and #3
#5 randomized controlled trial[pt]
#6 controlled clinical trial[pt]
#7 randomized[tiab]
#8 placebo[tiab]
#9 drug therapy[sh]
#10 randomly[tiab]
#11 trial[tiab]
#12 groups[tiab]
#13 #5 or #6 or #7 or #8 or #9 or #10 or #11 or #12
#14 animals[mh] not humans[mh]
#15 #13 not #14
#17 #4 and #15

### 2.4 Study selection and data extraction

**Figure 1:**
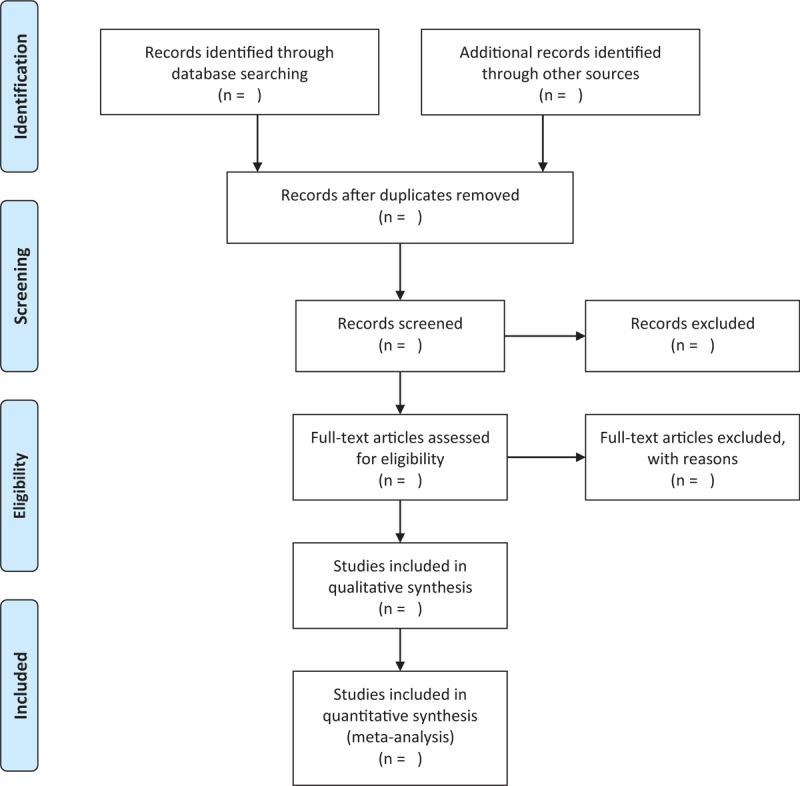
The PRISMA flow chart of the selection process.

First, titles and/or abstracts of studies retrieved using the search strategy and those from additional sources were screened independently by two review authors to identify studies that meet the inclusion criteria. Second, the full texts of these eligible studies were retrieved and evaluated independently by two authors based on the eligibility criteria. Any disagreements were resolved through discussion with a third reviewer. A flow diagram will be used to describe the selection process of eligible papers (Fig. 1). Data will be extracted through the pre-designed form from the included RCTs, which includes study population, age, gender and baseline characteristics, details of the intervention and control conditions, outcome measures and adverse events.

### 2.5 Risk of bias assessment

The methodological quality of the eligible studies will be evaluated according to the Cochrane Collaboration's tool for assessing the risk of bias, which considers random sequence generation, allocation concealment, blinding of participants and personnel, blinding of outcome assessors, incomplete outcome data, selective reporting and other sources of bias. Each domain will be assessed as “L”, “H” or “U” according to the description details of eligible studies, with “L” indicating a low risk of bias, “H” a high risk of bias and “U” an uncertain risk of bias.^[25,26]^ Two review authors independently will assess included studies. When disagreements exist, the problem will be resolved by discussion with the third review author.

### 2.6 Data synthesis and analysis

We will perform statistical analysis using Rev-Man version 5.3 software provided by Cochrane Collaboration (Copenhagen, the Nordic Cochrane Centre, the Cochrane Collaboration, 2014). Data will be summarized using risk ratios (RR) with 95% confidence intervals (CI) for binary outcomes or mean difference (MD) with 95% CI for continuous outcomes. For outcome variables on different measurement scales, we will use standardized mean difference (SMD) with 95%CI analyses. If the required data are not reported, we will request data from the corresponding author. We will use the fixed effects model unless there is evidence of heterogeneity to pool data across studies to conduct a meta-analysis. Heterogeneity will be assessed using both the χ^2^ test and the I^2^ statistic. We will consider an I^2^ value greater than 50% to be indicative of substantial heterogeneity, where a value of 0% to 40% is considered unimportant heterogeneity, 30% to 60% moderate, 50% to 90% substantial and 75% to 100% considerable. GRADE pro software from Cochrane Systematic Reviews will be applied to create a Summary of Findings table.^[27]^

### 2.7 Sensitivity analysis and subgroup analysis

Sensitivity analysis or subgroup analysis (conducted by Stata V.12.0) analysis will be performed if the heterogeneity or inconsistency among the studies is detected.^[28]^ We will perform a subgroup analysis based on the characteristics of the study to explore potential sources of heterogeneity, including sample size, asthma severity, treatment duration, and other relevant parameters. If the data extraction is not sufficient, the qualitative synthesis will be conducted.

### 2.8 Publication bias

A funnel plot will be generated to evaluate reporting bias when there are sufficient number of included studies. Egger tests will be applied to assess funnel plot symmetry and values of *P* < .1 will be as statistically significant. However, the funnel map asymmetry is not equal to publication bias, we aim to determine the possible causes of any asymmetry in the included studies, such as small study effects, poor methodological quality, and true heterogeneity.

### 2.9 Quality of evidence

The quality of evidence will be assessed for the main outcomes according to the Grading of Recommendations Assessment, Development and Evaluation approach (GRADE). Five items will be investigated, including limitations in study design, inconsistency, inaccuracies, indirectness, and publication bias.

### 2.10 Patient and public involvement

The patients and/or public will not be involved because this study uses secondary sources for analysis.

## 3 Discussion

To our knowledge, this is to date the most comprehensive meta-analysis that has evaluated efficacy and safety of YPFP for asthma in children. A systematic review of the immune effects of YPFP on asthmatic children has been published, but these studies excluded patients with cough variant asthma (CVA).^[29]^ Our study does not only include CVA subjects, but also the diagnosis on asthma in children is based on the Chinese Guidelines for the Prevention and Treatment of Asthma in Children or GINA Guidelines. We excluded those patients who were not diagnosed by doctors to avoid expanding the efficacy of YPPF. Similarly, to make the results more reliable and convincing, the outcome indicators are also based on the Chinese Guidelines for the Prevention and Treatment of Asthma or the Guidelines for Clinical Research of New Chinese Medicine. In addition, children with asthma of various severities were included, which reflects that YPPF is applied more expansive and more applicable to current clinical practice in many ways.

However, there are also limitations to this study which need to be considered. It may not be possible to extract all outcomes for each trial included, which will reduce the strength of evidence when analyzing these particular outcomes as it is possible that certain comparisons may not be able to be estimated from the network. Moreover, some gray literature may be sometimes difficult to retrieve, possibly leading to a selection bias in the literature. Nevertheless, we plan to conduct the research in order to provide useful information to practitioners and patients with asthma. Hopefully, it can be valuable adjuvants to improve the efficacy of asthma in children and reduce the side effects of the corticosteroids.

### 3.1 Ethics and dissemination

Formal ethics approval is not necessary for this study as confidential patient data will not be analyzed. The results of the meta-analysis will be communicated according to the PRISMA extension statement and disseminated in a peer-reviewed journal.

## Author contributions

Contributors to the conception, design and/or interpretation and drafting of this systematic review and network meta-analysis protocol: Ruiyin Wang, Jianxin Wang, JunShu. Xianmin Gu and Hongwen Li searched the literature and performed data analysis. Yingxin Zi and Shufang Liu revised it. Approving the final version of the protocol: Jiangtao Lin. All authors have approved the final manuscript.
